# JS-MS: a cross-platform, modular javascript viewer for mass spectrometry signals

**DOI:** 10.1186/s12859-017-1883-6

**Published:** 2017-11-06

**Authors:** Jebediah Rosen, Kyle Handy, André Gillan, Rob Smith

**Affiliations:** Department of Computer Science, University of Montana, 32 Campus Drive, Missoula, 59812 MT USA

**Keywords:** Mass spectrometry visualization, Mass spectrometry JavaScript, 3-D visualization, Mass spectrometry data processing, JavaScript mass spectrometry parsing

## Abstract

**Background:**

Despite the ubiquity of mass spectrometry (MS), data processing tools can be surprisingly limited. To date, there is no stand-alone, cross-platform 3-D visualizer for MS data. Available visualization toolkits require large libraries with multiple dependencies and are not well suited for custom MS data processing modules, such as MS storage systems or data processing algorithms.

**Results:**

We present JS-MS, a 3-D, modular JavaScript client application for viewing MS data. JS-MS provides several advantages over existing MS viewers, such as a dependency-free, browser-based, one click, cross-platform install and better navigation interfaces. The client includes a modular Java backend with a novel streaming.mzML parser to demonstrate the API-based serving of MS data to the viewer.

**Conclusions:**

JS-MS enables custom MS data processing and evaluation by providing fast, 3-D visualization using improved navigation without dependencies. JS-MS is publicly available with a GPLv2 license at github.com/optimusmoose/jsms.

## Background

**Fig. 1:**
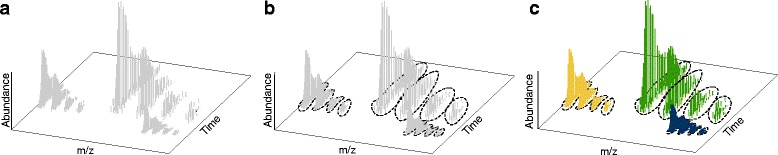
In a mass spectrometry experiment (**a**), each unique molecule in a sample will create a unique 3-d signal group, called an *isotopic envelope* (indicated by color in (**c**)), for every charge state found in the analysis. Each isotopic envelope is comprised of several isotope traces (indicated by dashes in (**b**))

Mass spectrometry (MS) plays a role in many biological/biomedical investigations [1] because it can quantify and identify the major components (proteins, lipids, metabolites) of most cellular systems. MS is a long-standing technology that is vital for answering a variety of experimental questions across many disciplines [2]. MS yields a list of identities and quantities of molecules in a sample through the analysis of signals generated by charged molecules (see Fig. 1).

While fragmented spectra are of principle interest in current proteomics mass spectrometry workflows, precursor or MS1 data is important in proteomics and other mass spectrometry applications due to its usefulness in constraining the peptide spectra search space, providing quantitative information, and in workflows (e.g. metabolomics or lipidomics) where fragmentation rules are not well known.

In such cases, there exists a need to view and interact with MS1 data. The most appropriate open source software for this purpose is TOPPView [3], a module of OpenMS. TOPPView opens any.mzML file and displays it in a 3-d representation with zoom and rotate capabilities. Repurposing TOPPView for manual MS data processing reveals several shortcomings:

- TOPPView automatically filters out some visible points, making it impossible for the practitioner to dictate how these points ought to be interpreted.
- The only way to annotate data in TOPPView is to export the visible subset of data as an.mzML file. The exported data will include hidden points, and further processing requires programming expertise to process the exported data.
- TOPPView’s data plot does not allow for shifting to the right, left, up, or down. In order to shift the view, the user must zoom out and carefully zoom back in adjacent to the previous area. This makes navigation difficult, and keeping track of the unprocessed portion of the data exceedingly difficult.
- TOPPView requires the installation of the large OpenMS library. This is not an issue on Windows or Mac, but for linux requires the installation of several difficult to install dependencies and a local build, which is beyond the technical ability of many potential users.

The form and function of current user interfaces do not provide the tools necessary to automate the user’s involvement, nor the visualizations necessary to efficiently iterate through the data.

In this manuscript, we present JS-MS, a 2- and 3-D MS viewer designed to as an intuitive, fast, specialized MS viewer. JS-MS allows users to better study data characteristics to design and test MS data processing algorithms. JS-MS is implemented in JavaScript. It is lightweight, has no dependencies, is cross-platform, and provides all of the viewing features of TOPPView without filtering points, plus the ability to track (move up/down/left/right without adjusting zoom).

JS-MS is designed under a modular view paradigm. It responds to a simple JSON API, making it extensible to any workflow. JS-MS is packaged with a modular back-end implemented in Java as an example of how it can operate modularly with any backend via a well-defined API. The package is provided as a single self-contained JAR file so that the only prerequisites to run the software are the Java Runtime Environment (JRE) and a web browser, typically pre-installed on any computer.

In this paper, we discuss the design of JS-MS and demonstrate its capabilities. JS-MS is publicly available and has been tested on Windows, Mac, and Linux operating systems with Chrome, Firefox, and Edge browsers.

## Implementation

JS-MS is a cross-platform graphical interface for viewing and segmenting mass spectrometry data. Existing MS viewers often require the installation of external packages which can pose an onerous burden on the user, particularly for Linux users who might have to compile required external libraries. JS-MS is a 3-D MS viewer which is self-contained and cross-platform. The JS-MS viewer itself is implemented via HTML and JavaScript alone. It runs as a web application without the need for any external dependencies by using WebGL [4], a 3-D graphics library included with all major modern browsers.

### View

**Fig. 2:**
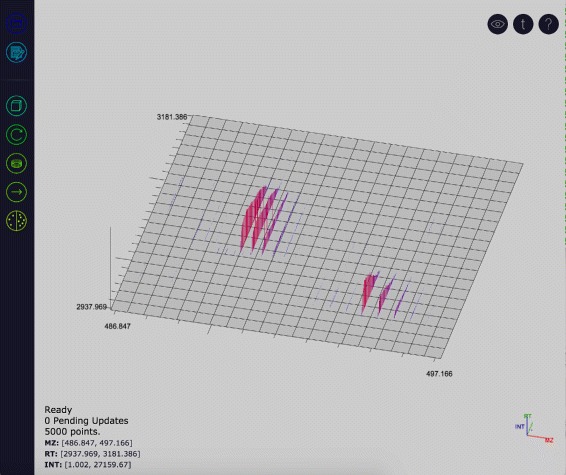
The JS-MS client. The toolbar (left) allows for data manipulation and navigation. The legend (bottom right) indicates the current view in relation to the data set. The graph can display the data in two-dimensional or three-dimensional mode (shown)

JS-MS is composed of a graph view rendered using the three.js [5] JavaScript graphics library and a button toolbar (see Fig. 2). The graph view is a three-dimensional graph displaying mass-to-charge (m/z), retention time (RT) and intensity dimensions. The graph layout follows standard conventions, with m/z and RT comprising the horizontal *x* and *y* axes respectively, and intensity plotted on the vertical axis. The graph can be zoomed, scrolled, and rotated, allowing full navigation of even the largest MS data sets.

### Data rendering

Web browsers are attractive targets for user interfaces due to the flexibility of HTML and CSS, but JavaScript performance can be an obstacle. The most influential component of memory usage and performance is rendering load, that is, how many points are drawn on screen. Almost every MS data file contains too many points to be rendered at once. Although system memory can load more points than can be rendered at once, RAM also becomes a secondary ceiling that must be addressed, though this is a problem that is handled by the backend and outside the scope of JS-MS.

An effective representation of the original full data set at any granularity is essential to maintaining a small rendering load while maintaining an accurate representation of the underlying data. Ideally, representations would be selected in such a way that the points chosen are real data points, that is, they are not an aggregation of points, and that the resulting graph looks similar to a graph of all original points within the viewing area. The first requirement is necessary for performing data processing, and the second requirement makes a great difference in usability and navigability of the data for the end user. The point selection process is referred to as “summarization”. While summarization takes place in the data store module and not JS-MS, a brief description of efficiencies designed into MzTree [6]–JS-MS’s default data store module–is useful to understanding JS-MS’s design and performance.

The MzTree data structure performs summarization both statically and dynamically.

Static summarization occurs at MzTree construction time. First, the data set is culled to all points above an intensity threshold of 1–points with intensity below 1 are considered noise. This typically reduces data cardinality by over fifty percent. Second, MzTree is built recursively into a hierarchical bounding boxes. Each leaf of the MzTree contains data points, and each internal node samples a preset number of points from the set of all child nodes’ points. Each sample is merely a collection of pointers to avoid data duplication. This process has a trickle-up effect starting at the MzTree leaf level (all data); each level of the tree contains a sample set of points with a degree of summarization equal to its height. After construction completes the resulting MzTree has numerous prepared data samples of ascending detail levels for any given viewing area.

Dynamic summarization occurs at runtime on a per-query basis. Upon receiving a query (consisting of a *viewing area* and *desired number of points*) the MzTree is traversed breadth-first starting at the root node. At each level in the tree, each node’s data bounds are compared with the query’s *viewing area*. If the node’s data bounds overlap with the query’s *viewing area* its within-view points are included in the prospective point set for that level in the tree. The size of the prospective point set is then compared to the *desired number of points*. If the point set is smaller than the *desired number of points* the next level of the tree is processed (except at the leaf level, where fewer points than requested must be returned). If the level’s point set is sufficiently large dynamic summarization occurs on the point set to whittle it down to the *desired number of points*. Dynamic summarization enables query flexibility in the MzTree to ensure that JS-MS receives exactly the number of points requested.

While JS-MS is memory conservative by default, users have the option of increasing memory usage as a means of degrading render time. In the current implementation of the JS-MS viewer, a user-configurable level of detail determines the desired number of points per query. Detail level is implemented as a fixed number of points to render regardless of viewing area and location; by maintaining the same rendering load for any region of the data set, application performance remains uniform across an entire user session. Not all users have the same hardware capabilities or preferences for speed versus detail, thus the detail level is adjustable using a slider on the toolbar. In addition to the configurable detail level, JS-MS minimizes memory usage by keeping in memory only the currently visible data. Since the MzTree data server is exceedingly responsive, the viewer has no need to cache or prefetch data and can discard all loaded points from memory any time the viewing area changes.

## Results

JS-MS’s user interfaces and data rendering provide performance and functionality unavailable in other MS viewers.

### View

JS-MS is composed of a graph view rendered using the three.js [5] JavaScript graphics library and a button toolbar (see Fig. 2). The graph view is a three-dimensional graph displaying mass-to-charge (m/z), retention time (RT) and intensity dimensions. The graph layout follows standard conventions, with m/z and RT comprising the horizontal *x* and *y* axes respectively, and intensity plotted on the vertical axis. The graph can be zoomed, scrolled, and rotated, allowing full navigation of even the largest MS data sets.

**Fig. 3:**
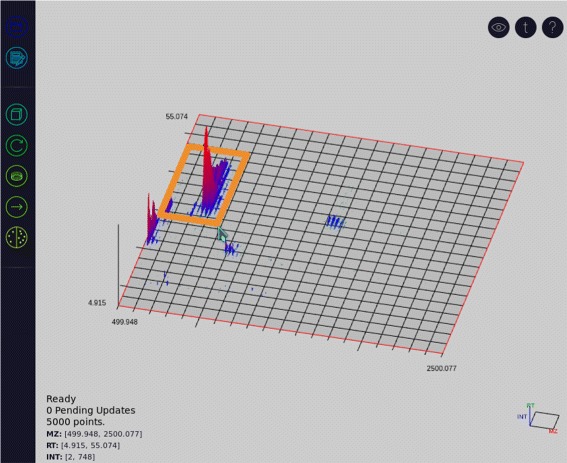
By using the mouse, a rectangular area can be set as the new viewing window in order to control the aspect ratio or focus on a specific section of the data set

The user is able to control the view range and perspective of the graph with a variety of graph transformations. Zoom level can be altered using one of two options: the mouse wheel, or a rectangular click and drag (see Fig. 3). The mouse wheel allows the user to zoom centered on the location of the mouse pointer. Alternatively, holding the control key activates click and drag zooming which allows the user to left-click and drag the mouse to specify a view window (see Fig. 3). In this manner rectangular zooming grants control over the graph’s aspect ratio.

JS-MS provides a rotation mechanism to allow users to view the data at different three-dimensional perspectives. By right-clicking and dragging, the graph can be rotated in any direction. Dragging vertically pitches the graph, dragging horizontally yaws.

The view range can be translated in all four horizontal directions by scrolling the graph. Scrolling is achieved by either left-clicking and dragging or using the arrow keys. Clicking and dragging scrolls the graph in the direction of the drag by the distance of the drag. Pressing an arrow key incrementally moves the graph in the key’s direction.

To further enable fast navigation, JS-MS is equipped with a jump to m/z feature. This feature allows the user to enter an m/z value and be immediately placed at that m/z position, while maintaining the current RT position, zoom level, and perspective.

To our knowledge, these features do not exist in any other 3-D MS viewing software. Without them, visiting each data point in a file would require zooming out and zooming in on adjacent data, a time-consuming practice resulting in loss of ones place and skipped points.

Two-dimensional mode provides a bird’s-eye view of the data. It is particularly useful for detecting low intensity points that would go unnoticed from a three-dimensional perspective. Switching to two-dimensional mode transforms data lines to squares so that they are visible from an overhead perspective. A variety of controls activate two-dimensional mode. Users can quickly view the data two-dimensionally from three-dimensional mode by either rotating to a bird’s-eye perspective or pressing and holding the shift key (releasing the shift key returns to the previous perspective). To persist the two dimensional view, the user can activate a toolbar toggle button to switch to two-dimensional mode. All applicable three-dimensional interfaces remain the same in two-dimensional mode, providing a seamless experience for the user. For example, the user can scroll the two-dimensional graph with the same controls as the three-dimensional graph.

### Data rendering

.mzML is the standard open, vendor-neutral data format for storing and communicating mass spectrometry data and follows the XML schema [7]. Parsing an.mzML file to extract all MS data points is necessary for using JS-MS, for using any other three-dimensional viewer, or for converting to novel file formats that are not spectrum ordered. This requires a new approach to reading.mzML files that is both fast and memory efficient.

There are mature libraries for parsing mzML files in many languages, such as C++ and Python; however, options for parsing.mzML files in Java are limited. Jmzml, an existing library for processing.mzML files in Java, is optimized for memory-efficient processing of large files. It implements on-the-fly indexing of files to ensure only requested data is loaded into memory [8]. Jmzml is optimized for multiple, fast reads after a lengthy index-rich initialization, making it poorly suited for parsing.mzML to convert into another data structure. DOM (Document object model), a popular API for interacting with XML documents, is very fast when parsing large files but uses considerable memory (several times the size of the XML file). Since.mzML files can run into the tens of GBs, this is a limiting feature of DOM.

A single pass parser using the StAX (Streaming API for XML) interface in Java provides fast MS data extraction with minimal memory usage.

.mzML parsers based on the StAX API, the DOM API and Jmzml were implemented for reading the set of points in an.mzML file. The parsers were compared based on memory footprint and execution time. Experiments were carried out on a set of.mzML files from several MS experiments. File size varied from 4.3 to 1240 MB and included indexed and non-indexed.mzML files. Both uncompressed and zlib compressed binary arrays were used. Testing was performed on a personal computer running Ubuntu with an SSD. For timing experiments, minimum and maximum Java heap space were both set to 4 GB. Memory footprint was measured by reducing the Java heap space allocated until the parser failed to execute. The parsers were modified to discard points after accessing them in order to give an accurate measurement of the parser’s share of total memory.

Overall execution time was measured for all tests; in addition, build time for the parser object and actual parse time were measured for each of seven files by placing timers inside the code for each parser.

For each of the input.mzML files above 50 MB, a set of files containing subsets of the spectra for that original.mzML file were generated. For instance, for a file with 1000 spectra, a file containing only the first 20 of these spectra, a file containing the first 40 spectra, and so on could be created to yield a total of 50 files. In this way, the execution time for parsing as a function of file size or number of points was measured, with other aspects of the file held constant [9].

**Fig. 4:**
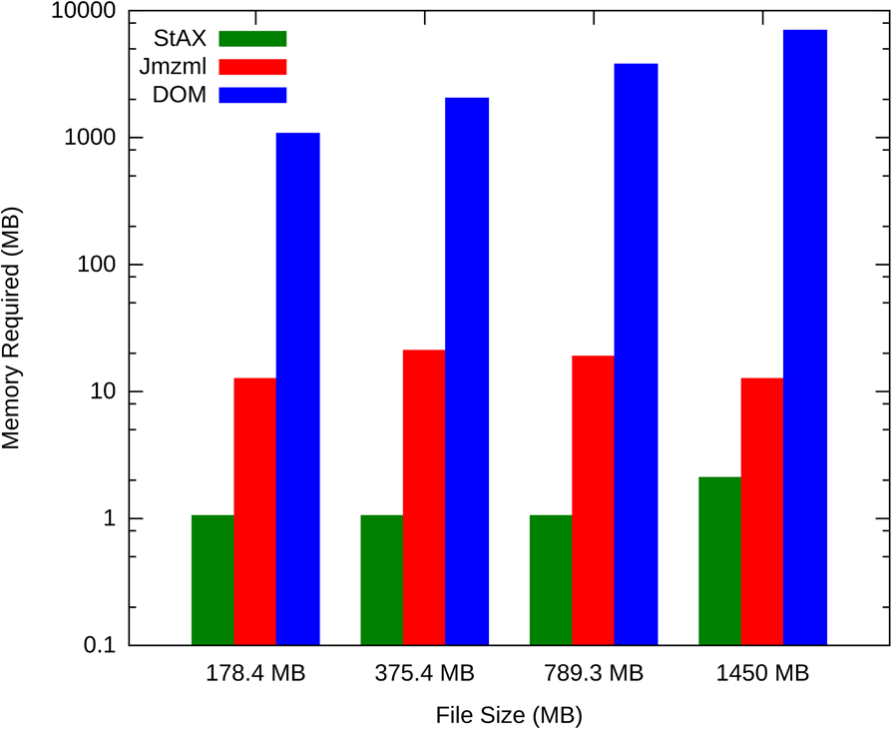
Parser object memory usage for mzML files of different sizes. Required memory is shown on a logarithmic scale. StAX and Jmzml parsers both use significantly less than DOM

Memory usage of the different parsing objects in shown in Fig. 4. DOM parsing requires memory for indexing overhead that while amortized for larger file sizes, still leads to much greater requirements than Jmzml, which uses roughly two orders of magnitude less, and StAX, which uses about two orders of magnitude less. StAX memory requirements do not increase noticeably even on large files.

**Fig. 5:**
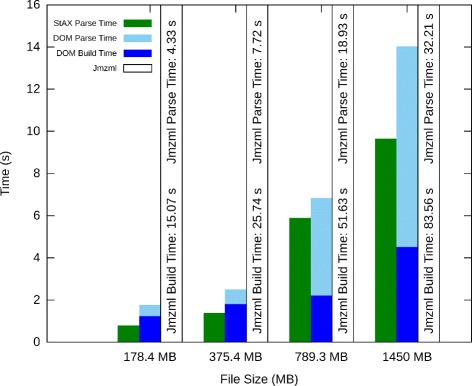
Parser object build time and parsing time shown separately for several different file sizes. For Jmzml, build time is significant, while for DOM build time is small and for StAX it is negligible

Figure 5 shows build time and parse time separately for the different parser objects. StAX outperformed DOM on all tests, with Jmzml requiring performing much slower than either alternative. under normal circumstances. For DOM, build time was minimal, and for StAX, build time was negligible.

**Fig. 6:**
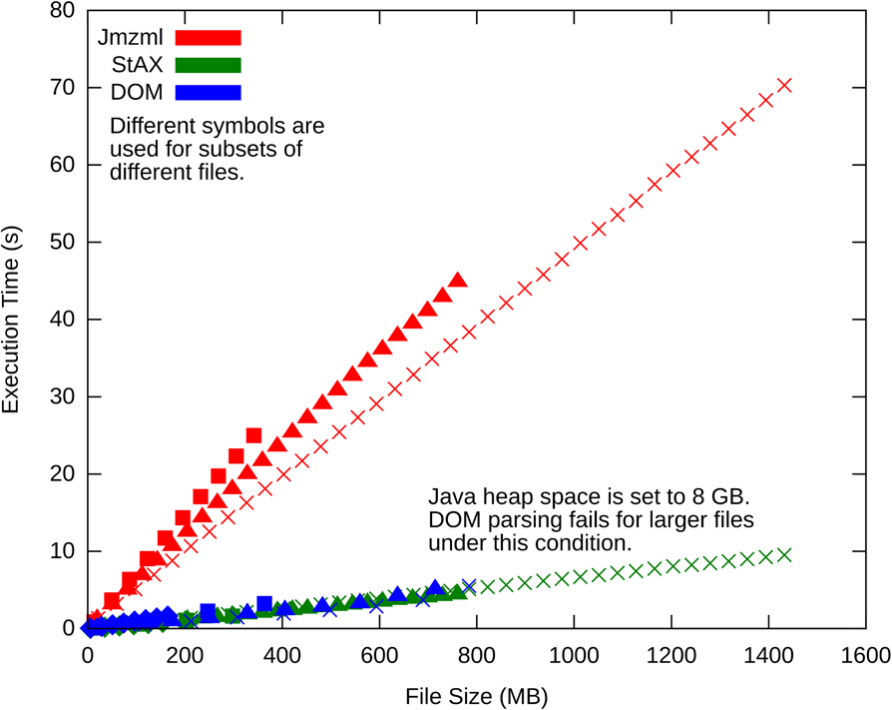
Execution time versus file size for the three parsing models and for spectra subsets of multiple files. Execution time versus file size is linear in this range for the same parser along subsets of the same file. StAX and DOM perform similarly and always outperform Jmzml

Figure 6 shows plots of execution time versus file size for spectra subsets. The plots are linear for all parsers. This is to be expected, as file reading is a linear algorithm. For Jmzml, execution time increased more quickly with file size than for DOM and StAX, implying that Jmzml will always be slower even as the file size increases. This is likely because Jmzml has to read the file from the disk twice–once to index the file and once to access the data–while DOM and StAX only have to read the file once.

## Discussion

Performance-centric design allows JS-MS to handle the overwhelming processing and storage demands for a web environment created by the magnitude of mass spectrometry data. The viewer logic is fully contained in a thin client designed to flexibly interface with any data storage and retrieval system that interfaces with the JS-MS HTTP API. Our implementation is a Java web server that is designed to run either on the same machine as the client, or as a standard stand-alone server. The viewer is responsible for drawing the current window of data provided on the latest request to the server. The decoupling of the viewer from the backend allows implementation and testing of various different approaches for data storage and retrieval, algorithmic data processing, and manual inspection.

JS-MS provides two main advancements: First, it is the only JavaScript viewer for MS data. Through the several optimizations discussed above, JS-MS is able to quickly plot 3-D MS data despite being implemented in JavaScript, giving it browser-based cross-platform compatibility, with access to virtually unlimited extensibility provided from the wide array of tools available in lightweight JavaScript libraries. Second, it implements critical navigation features unavailable in other MS viewers. These features, such as scroll and jump to m/z, allow users to inspect adjacent signals without needing to zoom out and zoom in on an adjacent area–a very difficult task given the self-similar nature of MS data. Another feature, the instantaneous toggle between 2-D and 3-D mode, is absent from other popular viewers, such as TOPPView, which requires another view window and navigating to the same spot.

JS-MS is modular because it makes no assumptions about previous or successive data processes, other than what is defined in the API. You can use any backend of choice with JS-MS, and use the data for any downstream processes. JS-MS’s included Java server provides a working example of how users can interact with the viewer using its HTTP API, and also is a stand alone contribution for those who want to implement their own modular data processing tools for MS. The StAX parser for.mzML, which outperforms other publicly available parsers, should be useful for anyone who is using MS data with Java code. Those who wish to implement their own server or use another pre-existing server can do so provided they implement the API that JS-MS expects.

## Conclusion

JS-MS is a cross-platform, modular, browser-based MS data viewer. It runs on any modern browser without additional dependencies. It is the first MS viewer to provide a full suite of navigational tools (including scroll). JS-MS’s advanced interfaces and novel backend enable sufficient speedup to make visual analysis practical.

## Availability and requirements

JS-MS is implemented in JavaScript and Java, runs on all operating systems, and is available with a GPLv2 license from github.com/optimusmoose/jsms.

## Abbreviations

MS: Mass spectrometry
m/z: Mass to charge ratio
RT: Retention time
